# Clinical and cytogenetic analysis of human anemias from Jammu region of Jammu and Kashmir state

**DOI:** 10.4103/0973-6247.67031

**Published:** 2010-07

**Authors:** Upma, Parvinder Kumar, T. R. Raina, Kuldeep Sharma, Subash Gupta

**Affiliations:** Institute of Human Genetics, University of Jammu, Jammu- 180006, India; 1 Department of Blood Transfusion & Immunohaematology Med., Govt. Medical College, Jammu, India; 2 Department of Zoology, University of Jammu, Jammu-180006, India

**Keywords:** Anemia, centromere stretching, chromatid breaks, chromosomal aberrations, megaloblastic anemia

## Abstract

**Background:**

Anemias are the blood disorders characterized by reduction in the number of circulating red blood cells, the amount of hemoglobin, or the volume of packed red cells in blood. Chromosomal aberrations have often been reported from the bone marrow as well as cultured lymphocytes of the anemic patients.

**Aims:**

The aims of the study were to find out the commonest type of anemia occurring in the population of Jammu, India and to find out the chromosomal changes involved in the disorder.

**Material and Methods:**

Present study has been carried out on the bone marrow samples from 53 clinically diagnosed anemic patients. Cytogenetic study was carried out on slides prepared from these samples. Noncytogenetic factors like age, sex, religion, blood groups, family history of anemia, socioeconomic status, etc. have also been included in the study.

**Results:**

Megaloblastic anemia was found to be the commonest type of anemia. Centromere stretching, chromatid breaks, gaps, and elongation of chromosomes were recorded in patients with megaloblastic anemia and combined deficiency anemia. However, structural changes and numerical changes were totally absent.

**Conclusion:**

The commonest anemia among the people of Jammu region is megaloblastic anemia and its prevalence is increasing every year. Also, megaloblastic anemia is always associated with reversible cytogenetic changes.

## Introduction

Anemia is a common problem in primary healthcare practice, occurring with an annual incidence of 18 cases per 1,000 patients.[1] It is estimated that 10% of the population in the developed countries and as much as 25–50% in developing countries are anemic.[2] Anemias are of several types; however, megaloblastic, iron deficiency, sideroblastic, hemolytic, and aplastic anemia are the commonest. Microcytic anemia is the most common form of anemia in children and adolescents. It is a very heterogeneous group of diseases that may be either acquired (mostly due to iron deficiency) or inherited. In recent years, human patients or animal models have highlighted the existence of new conditions involved in the pathogenesis of hereditary microcytic anemia.[3] Anemia is common in older adults, and the prevalence of anemia increases with advancing age.[4,5] Studies have been carried out from time to time to find out the cytogenetics of different types of anemias, and it has been found that not all but many of them are associated with chromosomal changes. There have been reports of aneuploidy[6,7] and loss of Y chromosome[8] in case of sideroblastic anemias; trisomies of 6 and 8 and loss of chromosome 7[9] in aplastic anemia. Association of reversible cytogenetic abnormalities with megaloblastic anemia have also been observed.[10] The present study was conducted to find out the commonest types of anemias occurring in the population of Jammu, Jammu & Kashmir, and to find out the chromosomal changes involved in the disorder.

## Materials and Methods

Fifty-three cases with the history of anemias referred to the Department of Pathology, Government Medical College (GMC), Jammu, were taken up for the cytogenetic studies. Work has been carried out in Human Genetic Research cum Counselling Centre, University of Jammu/GMC, Jammu, India Ethical clearance was obtained from competent authority. *In vivo* chromosome study was carried out from the bone marrow that was aspirated in routine for the hematological findings. A few bits of the bone marrow in each case were collected in hypotonic solution 0.075 M KCl and these bits were agitated so as to make cell suspension. This was kept in the hypotonic solution for 35–45 minutes at room temperature. Cell suspension was then fixed in 3:1 methanol and acetic acid and the same was subsequently processed for the chromosome study, following Miles *et al*.[11] with slight modifications. About 7-days old slides were G-banded following Sea bright[12] with slight modifications. G-banded metaphase plates were analyzed for the study of chromosomal aberrations.

On the basis of hematological reports, 53 cases were divided into four categories i.e. megaloblastic anemia (28); combined deficiency (megaloblastic changes + low iron stores, no: 7); iron deficiency (12) and normoblastic erythropoiesis (6).

## Results

Of the 53 cases in the present study, male to female ratio was 1.12:1. The hemoglobin levels ranged from 2.0 to 9.8 g/dl, the age group ranged between 21–30 and 41–50 years. Majority of the patients were Hindus (86.79%). Most of the cases had a rural background (64.15%) and B+ve blood group was found to be the commonest one (39.6%).There was no family history of anemia in the 53 cases. Majority (75%) were non-smokers and non-alcoholic, while the remaining 25% were either smokers, alcoholic, addict to drugs, or tobacco chewers. Megaloblastic anemia was more prevalent than any other anemia in the present study, that is, 52.83% (28 out of 53). Iron deficiency anemia was the next common with 12 out of 53 patients (22.64%) as sufferers. Normal diploid chromosome number 46 was recorded in all the 53 cases. Hypodiploidy was seen only in a few cases and it was less than 10%; however, changes in chromosome morphology were seen in all the cases of megaloblastic anemia and those with combined deficiency. These changes were as follows:

1. *Chromosome breaks and gaps :* Of the 28 patients with megaloblastic anemia, breaks and gaps were observed in 22 cases (78.57%). The percentage of scorable cells with breaks and gaps in metaphase chromosomes was 4.8–43.5% and individual cells showed several breaks. In patients with combined deficiency, breaks and gaps were observed in all the seven cases. About 3.8–37.5% of the scorable cells showed breaks and gaps in the metaphase chromosomes.
2. *Centromere stretching :* It was the commonest of all abnormalities being present in 10–66.6% of the scorable cells in all the 28 cases of megaloblastic anemia and in 2–30.8% of the scorable cells in 6 cases of combined deficiency anemia.
3. *Centromere spreading :* It was observed only in one case of megaloblastic anemia in which only 13% of the scorable cells showed spreading of centromere. In all the other cases, centromere spreading was totally absent.
4. *Chromosome elongation :* A number of cells in all cases of megaloblastic anemia as well as that of combined deficiency showed thin elongated chromosomes, which probably resulted either from reduced contraction or despiralization.

In cases with iron deficiency and those with normoblastic erythropoiesis, no changes either in chromosome number or morphology were observed.

## Discussion

Megaloblastic anemia is not uncommon in India, but data are insufficient regarding its prevalence, causative, and precipitating factors. The prevalence of megaloblastic anemia was found to be 52.83% in our study sample, while it has earlier been reported to be 42.1% by Gomber *et al*.[13] and 27.1% by Chaudhary.[14] Since, the prevalence of megaloblastic anemia in the present study is higher than the earlier reports, it supports the earlier findings[15] that reported an increase in the prevalence of megaloblastic anemia over the years. The peak incidence of megaloblastic anemia had been recorded earlier in the age group of 10–30 years.[16] However, in the present cases, it was in the age group of 31–50 years. Therefore, the present findings on the correlation of age group with megaloblastic anemia are different from the existing reports.

In alcoholics, megaloblastic anemia develops more frequently than any other type of anemia.[17] But the study showed a different picture as megaloblastic anemia has been found only in 20% of the alcoholics, while iron deficiency was found in 40% of them. Rest 40% showed normoblastic erythropoiesis. The changes in morphology of the chromosomes in megaloblastic anemia especially centromere stretching, chromatid breaks, and incomplete contraction are in line with those of the earlier workers. Centromere spreading could be recorded only in one case of megaloblastic anemia, while earlier workers have observed centromere spreading in megaloblastic anemia as the most common morphological abnormality of chromosomes.[18] In the present study, the frequency of anemic males compared to females was higher. Similar findings have been reported by Yoshida *et al*.[19], Ilgin *et al*.[20], and Maciejewski *et al*.[21]

## Conclusion

The clinical and cytogenetic aspects of human anemias were studied in 53 cases having history of anemias by examining the direct marrow preparations. More males were found to be suffering from anemias as compared to females. Megaloblastic anemia was more prevalent than any other type of anemia. Morphological changes in chromosomes were observed in all the patients with megaloblastic anemia and combined deficiency anemia. No numerical changes, translocations, and chromatid exchanges were observed in any of the cases. Centromere stretching, chromatid breaks and gaps, and elongation of chromosomes were the common morphological chromosomal abnormalities, while centromere spreading which is a common feature of megaloblastic anemias was almost absent. The karyotypes of all these patients were normal with 46XX/ 46XY.
